# Postconceptional and Hereditary Syphilis

**Published:** 1913-08

**Authors:** 


### ABSTRACTS.

---

POSTCONCEPTIONAL AND HEREDITARY SYPHILIS.—Jean Bobrie (*Etude sur la Syphilis*, etc., Paris, 1912) gives the following conclusions from his study of postconceptional syphilis and heredity of syphilis. In the absence of all treatment we may find placentas that are macroscopically healthy along with children evidently syphilitic. Nevertheless, maternal treatment has a marked effect on placental hypertrophy. The gravity of the syphilis of the fetus bears no relation to the hypertrophy of the placenta. Hydramnios is exceptional. At whatever time of pregnancy the maternal chancre is contracted the fetus is still infected. The degree of infection varies with the time of infection; the maximum gravity, causing macerated fetus, is at the third month; the gravity then decreases. The fetus never receives a true immunity; while there may be no evidence of syphilis the disease is still present in a latent form. Postconceptional syphilis is more serious for the existing pregnancy than for a later one. The fetus is always infected long before the roseola appears; infection occurs as soon as the mother has been attacked by the chancre. The most effective treatment is mercury in the form of soluble salts or pills; gray oil and salvarsan have not so good an effect by far. Hereditary syphilis can only be transmitted to the fetus by way of the placenta; the spermatozoon which contains a treponema cannot fecundate an ovum. Exceptions to Colles' law are not true. Mothers who do not show syphilitic lesions are still infected, the syphilis being latent. Heredodystrophy is a consequence of syphilis, but not syphilis itself; such infants can become infected by syphilis, and it is then transmissible by the germinative cell only.—*Arch. of Pedeatrics,* May, 1913.

